# Draft Genome Sequence of *Pseudomonas pachastrellae* Strain CCUG 46540^T^, a Deep-Sea Bacterium

**DOI:** 10.1128/genomeA.00136-17

**Published:** 2017-04-06

**Authors:** Margarita Gomila, Magdalena Mulet, Jorge Lalucat, Elena García-Valdés

**Affiliations:** a Microbiologia, Departament de Biologia, Universitat de les Illes Balears, Islas Baleares, Spain; b Institut Mediterrani d’Estudis Avançats (IMEDEA, CSIC-UIB), Palma de Mallorca, Spain

## Abstract

*Pseudomonas pachastrellae* strain CCUG 46540^T^ (KMM 330^T^) was isolated from a deep-sea sponge specimen collected in the Philippine Sea at a depth of 750 m. The draft genome has an estimated size of 4.0 Mb, exhibits a G+C content of 61.2 mol%, and is predicted to encode 3,592 proteins, including pathways for the degradation of aromatic compounds.

## GENOME ANNOUNCEMENT

*Pseudomonas pachastrellae* strain CCUG 46540^T^ (KMM30^T^) was isolated from a deep-sea sponge (*Pachastrella* sp.) in 1991 and in 2005 was proposed as the type strain of this species (1). It is affiliated with the *P. pertucinogena* phylogenetic group, as established by Mulet and collaborators (2). *P. pachastrellae* is a strictly aerobic mesophilic species that tolerates up to 10% NaCl.

The whole-genome shotgun sequence of *P. pachastrellae* was performed using pair-end sequencing on the MiSeq platform system (Illumina). The Newbler Assembler version 2.7 software package (Roche) was used for *de novo* genome assembly. The draft genome size is 3,920,464 bp and contains 119 contigs, with an average contig length of 32,945 kb, a median coverage depth of 160×, and an average G+C of 61.2 mol%.

Prediction and annotation of the genome was performed using the NCBI Prokaryotic Genome Annotation pipeline (http://www.ncbi.nlm.nih.gov/genome/annotation_prok). Analysis and comparison of the functional annotation was done using the KEGG Automatic Annotation Server (KAAS) (3).

The genome is one of the smallest in the *Pseudomonas* genus. A total of 3,656 coding sequences, 54 tRNA sequences, and one rRNA sequence were identified in the chromosome. Flagellation and twitching motility genes have been found. Secretion systems of types I, II, and VI were localized in the genome, together with Sec-independent protein translocases. A complete set of genes was detected for the upper and lower degradation pathways predicted to catabolize benzene and toluene. A multicomponent phenol hydroxylase produces catechol that is metabolized by a lower meta-cleavage pathway to central metabolites. Genes were predicted for the assimilation of nitrate and nitrite as nitrogen sources (nitrate, nitrite, and ammonium transporter systems, as well as nitrate and nitrite reductases with their regulators), for a urea transport system, and for urease synthesis (although the strain was described as urease negative). Genes for catalase/peroxidase, for an iron complex transport system, and for an NADPH-dependent ferric siderophore reductase were also predicted. A complete set of genes was found for the synthesis of polyhydroxyalkanoate as a storage compound. CRISPR-associated proteins and a putative toxin/antitoxin system were annotated. No plasmids were detected, but the mobilome was characterized by 41 proteins annotated as transposases, some of them related to the IS5, ISL3, Tn3, and Tn5 families. Moreover, 1,150 encoded proteins were annotated as hypothetical proteins without a predicted function.

The three closest species to *P. pachastrellae* in the *P. pertucinogena* phylogenetic group (*P. aestusnigri*, *P. oceani*, and *P. sabulinigri*) have a marine origin—some from the deep sea, such as *P. oceani* (4). *P. pachastrellae* strains have also been found in polluted samples after a marine oil spill (5), like strains of *P. aestusnigri* (6), the phylogenetically closest species in the group. The sequenced genome of *P. pachastrellae* CCUG 46540^T^ will help to elucidate the phylogeny of the species in this group and also unravel the metabolic mechanisms shared by these microorganisms for survival in these environments.

### Accession number(s).

This whole-genome shotgun project has been deposited in DDBJ/ENA/GenBank under the accession number MUBC00000000. The version described in this paper is the first version, MUBC01000000.
